# From inclusive leadership to thriving at work: the mediating and moderating roles of teaching efficacy and professional identity

**DOI:** 10.3389/fpsyg.2026.1803220

**Published:** 2026-05-19

**Authors:** Qingzhu Gao, Yuhan Li, Guangyan Zhang, Bo Wang, Marina Dzhumabaeva, Gulnaz Adylbek Kyzy

**Affiliations:** 1 School of Business Administration, Dongbei University of Finance and Economics, Dalian, China; 2 Business School, Southwest Jiaotong University Hope College, Chengdu, China; 3 School of Economics and Management, Kyrgyz State University named after I Arabaev, Bishkek, Kyrgyzstan; 4 Training Department, Heilongjiang Agricultural Economy Vocational College, Mudanjiang, China; 5 College of Education, Kyrgyz State University named after I Arabaev, Bishkek, Kyrgyzstan; 6 College of Education, Kyrgyz National University named after Jusup Balasa, Bishkek, Kyrgyzstan

**Keywords:** inclusive leadership, professional identity, teaching efficacy, thriving at work, young teachers

## Abstract

**Introduction:**

As a pivotal dimension of sustainable career development, employee thriving at work has garnered substantial scholarly and practical attention as a salient indicator of optimal psychological functioning in organizational settings. Extant literature has preliminarily established correlations between general leadership styles and employee thriving; however, the precise mechanisms and contextual contingencies through which inclusive leadership—characterized by openness, availability, and accessibility—fosters thriving at work among young university teachers remain inadequately theorized and empirically underexplored.

**Methods:**

This study integrates social cognitive theory with the socially embedded model of thriving to conceptualize a moderated mediation framework. Using questionnaire data collected from 755 young faculty members across Chinese universities, we examine the mediating role of teaching efficacy—a core belief in one’s instructional capability—in transmitting the influence of inclusive leadership to thriving at work, as well as the moderating function of professional identity in strengthening these relational pathways.

**Results:**

Our analyses reveal four key findings: (1) Inclusive leadership exerts a significant positive effect on thriving at work; (2) It also substantially enhances teachers’ sense of teaching efficacy; (3) Teaching efficacy partially mediates the relationship between inclusive leadership and thriving; (4) Professional identity not only amplifies the direct effect of inclusive leadership on teaching efficacy but also reinforces the strength of the indirect effect of inclusive leadership on thriving via teaching efficacy.

**Discussion:**

By contextualizing the thriving construct within the unique socio-cultural milieu of Chinese higher education, this study provides a nuanced theoretical account of how and when inclusive leadership cultivates thriving at work among early-career academics. These insights contribute meaningfully to the growing discourse on leadership and workplace well-being, while also offering actionable implications for academic institutions seeking to foster supportive environments that enhance both teaching and research performance.

## 1 Introduction

Young college teachers, defined as full-time educators under 40 in higher education institutions, play a crucial role in shaping tomorrow’s leaders. These individuals are highly educated and bring fresh perspectives and innovative ideas to the field of education (Menon and Meghana, 2021). They are responsible for advancing college development, nurturing talent, and fostering knowledge innovation (Lindstrom et al., 2022). However, recent research has shown that young college teachers face increasing pressure in teaching and research duties (Lee et al., 2022; Docka-Filipek and Stone, 2021). Some institutions have introduced policies such as “tenure track” and “up or go,” leading to heightened internal competition among young faculty members (Figlio et al., 2015). Many young teachers resort to “self-exploitation” tactics, such as working long hours, to meet these demands. This external pressure has resulted in physical and mental exhaustion, diminished academic autonomy, increased professional burnout, decreased work vitality, and even health issues (Mychajluk, 2024). Many prior studies have found that young teachers’ thriving at work can alleviate professional burnout, enhance job satisfaction and happiness (Tang et al., 2023), improve overall physical and mental well-being, boost teaching performance (Conway and Foskey, 2015), and contribute to overall academic success (Tobias et al., 2023).

Thriving at work refers to a psychological state in which young teachers experience vitality and a commitment to learning (Goh et al., 2022). The learning aspect encompasses the acquisition and application of new knowledge and skills, while the vitality aspect reflects a positive work experience characterized by enthusiasm and energy (Babalola et al., 2022). When vitality combines with learning, it can cultivate a healthy and motivating work environment, foster the development of teacher efficacy, and ultimately enhance organizational performance and success. Existing literature thoroughly analyzes the factors that contribute to thriving at work. For instance, research based on self-determination theory suggests that individuals’ behavior-such as task engagement, adventurous spirit, and psycho-logical capital-can significantly impact thriving at work (Kleine et al., 2019; Shen et al., 2024). Several studies indicate that external situational factors-such as organizational culture (Davenport, 2015), organizational support (Kleine et al., 2019), flexible decision making (Niu et al., 2024), information sharing and information quality (Jiang et al., 2024), and corporate climate and communication (Spreitzer and Hwang, 2019)- significantly influence thriving at work. However, existing research primarily focuses on individuals’ psychological and behavioral characteristics and organizational atmosphere, with relatively little attention paid to leadership’s impact on young teachers’ thriving at work.

College and university leaders are instrumental in fostering thriving. The perceptions of young teachers regarding organizational culture are heavily shaped by their leaders. Research indicates that leadership style significantly influences young teachers’ thriving at work (Niessen et al., 2017). Previous studies have explored the impact of transformational (Niessen et al., 2017), authentic (Durrah et al., 2024), and temporal leadership styles (Rahaman et al., 2022) on young teachers’ thriving at work. The socially embedded model of thriving at work emphasizes that employees who work in an environment characterized by open communication, mutual trust, respect, and high-quality work conditions tend to achieve greater levels of thriving at work (Spreitzer et al., 2005). As a distinctive feature of the work environment, inclusive leadership recognizes and appreciates the efforts and contributions of young teachers. It cultivates a sense of trust and respect among educators within the organization, actively welcomes innovative ideas from younger staff, and exhibits patience with their minor mistakes (Roberson and Perry, 2022; Shore and Chung, 2022). The inclusive leadership style addresses the psychological needs of young college teachers and has recently emerged as a prominent area of research within organizational behavior. The study found that inclusive leadership can boost young teachers’ work engagement, emotional commitment, and psychological safety, ultimately improving their thriving at work (Zhao et al., 2024). However, most studies on inclusive leadership and thriving at work are based on definitions and scales developed in Western countries. There is a lack of research that incorporates traditional Chinese culture and historical context to ex-amine how inclusive leadership styles, as defined within the Chinese context, influence young teachers’ thriving at work. This study aims to fill this gap.

Teaching efficacy refers to a teacher’s confidence in their abilities and their self-assessment of teaching effectiveness, which significantly impacts student learning outcomes (Ross and Gray, 2006). Social cognitive theory posits that the professional environment within a school plays a substantial role in shaping teachers’ sense of efficacy (Granziera and Perera, 2019). When teachers observe supportive behaviors within their organizational atmosphere, their teaching efficacy will be more effective. Observing a culture of support within their organization can boost teachers’ sense of teaching self-efficacy. For instance, enhanced support from school leaders, stronger relationships among colleagues, and a collaborative atmosphere can all contribute to increased teaching efficacy. Inclusive leadership plays a crucial role in shaping young teachers’ teaching efficacy. Leaders who embrace inclusivity show genuine concern for the work of these professionals, actively listen to their feedback, and are dedicated to fostering their professional growth. Moreover, inclusive leadership emphasizes the importance of respecting and addressing the reasonable needs of young teachers, which significantly enhances their teaching efficacy and revitalizes their teaching vitality. Accordingly, this paper will offer a comprehensive analysis of the mediating role that teaching efficacy plays in the relationship between inclusive leadership and young teachers’ thriving at work.

Professional identity is recognized as a significant attribute that has garnered considerable attention from researchers (Huy and Phuc, 2024; Chen et al., 2023). For young college teachers, professional identity encompasses a synthesis of their perceptions, experiences, and behavioral inclinations related to their profession and roles, constituting a favorable assessment of their vocation (Steinert et al., 2019). Existing literature indicates that professional identity is intricately linked to positive outcome variables, including professional commitment (Delima, 2015), professionalism (Colmer, 2017), engagement in work (Xing, 2022), and teaching efficacy (Rozati, 2017). Enhancing professional identity is beneficial for fostering individual professional growth and effectively mitigating turnover intentions. According to social cognitive theory, the development of professional identity among young teachers is a dynamic interplay be-tween individual cognition and the surrounding environment. Inclusive leadership functions as a key environmental mechanism in professional contexts, characterized by dimensions such as respect for varying pedagogical perspectives, acknowledgment of academic and professional distinctions, and cultivation of psychological safety. These components collectively constitute an ecological scaffold that shapes young teachers’ professional identity (Huy and Phuc, 2024). Teachers with a strong professional identity exhibit attributes like deep role identification, internalized professional values, a clear educational mission, and proactive commitment to instructional duties. Such traits enhance their responsiveness to inclusive leadership practices, enabling them to translate supportive environmental cues into motivational drivers for enhancing teaching efficacy. Thus, professional identity likely moderates the relationship between inclusive leadership and young teachers’ teaching efficacy. This study aims to incorporate professional identity into the framework of inclusive leadership and thriving at work, examining how the interaction between inclusive leadership and professional identity influences teaching efficacy and subsequent thriving at work.

Thus, drawing on social cognitive theory and the socially embedded model of thriving at work, this paper examines how inclusive leadership influences young teachers’ thriving at work, with teaching efficacy acting as a mediating variable. Additionally, this paper further introduces professional identity as a moderating variable, thereby examining the contextual boundaries within which this mediating mechanism operates. Using a sample of 755 survey responses from young college teachers, we apply SPSS 26.0, Process 4.1 macro and AMOS 29.0 to examine this relationship. This paper makes three significant contributions: First, it enhances our theoretical understanding of inclusive leadership by examining its impact on young teachers’ thriving at work within the Chinese higher education context. Our findings extend the theoretical reach of inclusive leadership to the Eastern context, thereby addressing the imperative for culturally-nuanced leadership research and establishing a critical empirical baseline for its global applicability. Second, exploring the mediating role of teaching efficacy in the relationship be-tween inclusive leadership and young teachers’ thriving at work, offers a valuable framework and perspective for understanding how inclusive leadership can foster young teachers’ thriving at work. Third, by introducing professional identity as a boundary condition, this study elucidates the contextual contingencies governing the impact of inclusive leadership on young teachers’ thriving at work. Crucially, we conceptualize professional identity not merely as an individual career commitment, but as a socio-cultural construct deeply rooted in the Chinese ethos regarding the moral stewardship of educators. Consequently, this research not only delineates the specific conditions under which inclusive leadership flourishes but also offers evidence-based insights for tailoring leadership interventions to culturally-specific organizational settings.

## 2 Literature review and hypothesis development

### 2.1 Inclusive leadership and young teachers’ thriving at work

Thriving at work refers to the psychological state in which individuals experience both “learning” and “vitality” in their professional lives (Goh et al., 2022; Kleine et al., 2019). “Learning” encompasses the ability to absorb and apply new knowledge and skills, while “vitality” signifies a state of active engagement, enthusiasm, and abundant energy (Niessen et al., 2012). These two dimensions are essential to thriving at work, reflecting the dynamic process of person-al growth. Individuals must experience learning and vitality concurrently to flourish and thrive at work (Xu and Wang, 2020). The teachers’ thriving at work is reflected in their commitment to continuous learning and professional development. The social embeddedness theory of thriving suggests that “vitality” and “learning” are deeply rooted in social systems, and that the social work environment shapes the thriving of college and university young teachers. Leadership style is a vital situational factor that significantly im-pacts young teachers’ thriving at work. The concept of inclusive leadership first emerged in education, where it referred to the inclusive management strategies employed by school leaders to address the diverse needs of learners (Roberson and Perry, 2022). Recent scholar-ship has begun to extend this notion to organizational management, defining inclusive leadership as the behaviors of leaders who actively encourage employee participation in decision-making and acknowledge the contributions of their team members (Nembhard and Edmondson, 2006). Some researchers describe inclusive leadership as a relational style prioritizing meaningful interactions between leaders and their subordinates (Carmeli et al., 2010). This style emphasizes attentive listening to employees’ opinions, sensitivity to their needs, and a commitment to openness, effectiveness, and accessibility. Inclusive leadership fosters a reciprocal relationship characterized by respect for individual differences (Shore and Chung, 2022). Leaders actively engage with their team’s needs and acknowledge the unique contributions of each member. Previous studies have shown inclusive leadership can significantly enhance young teachers’ work attitudes and emotional experiences, such as job satisfaction, trust, happiness, and professionalism (Babalola et al., 2022). Moreover, thriving at work is a positive psychological state that is significantly influenced by inclusive leadership within an organization. In other words, inclusive leadership encompasses a constructive approach rooted in mutual respect and equality (Shore and Chung, 2022; Ashikali et al., 2021). It exemplifies a dedication to recognizing and nurturing one another, fostering a supportive environment that empowers young teachers to thrive in their professional journeys.

First, inclusive leaders can encourage young teachers to participate in decision-making by cultivating an inclusive environment. These leaders actively listen to young teachers’ perspectives on college affairs, teaching conditions, and research initiatives, while embracing their diverse approaches and innovative ideas (Bao, 2024). Further-more, inclusive leaders demonstrate respect for individual differences and address mistakes made by young teachers with a sense of rationality and tolerance. This supportive environment meets the fundamental psychological needs of young educators, enhancing their interest and commitment to their teaching roles. Therefore, they be-come motivated to pursue higher aspirations, such as setting ambitious research goals, publishing high-quality papers, and mastering various research methodologies. Second, inclusive leaders who embody accessibility are approachable and genuinely invested in the well-being of young teachers from diverse subject backgrounds and varying levels of experience. They thoughtfully consider young teachers’ professional and personal lives in which they face challenges, concerns, and needs. Furthermore, inclusive leaders recognize and value the significant contributions of young teachers within the organization, fostering a positive leader-member exchange relationship (Wang and Shi, 2021; Ke et al., 2022). This high-quality relationship alleviates the uncertainties and work-related pressures that may impact the psychological well-being of young teachers in their teaching and re-search. It also enhances their sense of belonging within the organization and strengthens their professional identity. As a result, young teachers are motivated to demonstrate greater enthusiasm for their work and a deeper commitment to their roles, leading to increased job satisfaction and thriving at work. Third, inclusive leaders who demonstrate availability traits are instrumental in guiding young teachers as they face problems in teaching and research, especially when dealing with mistakes or setbacks. These leaders offer timely and professional guidance grounded in rational tolerance, fostering an environment emphasizing support rather than high-pressure control. Consequently, young teachers feel valued and receive the attention, encouragement, and training essential for their development. This nurturing atmosphere enhances their sense of organizational support and deepens their identification with the organization and commitment to its mission, motivating them to strive for meaningful goals and values. Hence, we hypothesize the following:

*H1*: Inclusive leadership positively relates to young teachers’ thriving at work.

### 2.2 Inclusive leadership and young teachers’ teaching efficacy

Teaching efficacy refers to teachers’ confidence in their instructional abilities, their self-assessment of those skills, and the impact these perceptions have on students’ learning outcomes (Kleinsasser, 2014). It relates to teachers’ internal beliefs about their ability to engage students and improve academic performance (Tschannen-Moran et al., 1998). Social cognitive theory suggests the work environment can significantly impact young teachers’ sense of efficacy (Wilson et al., 2020). Young teachers have a distinctly positive perception of the supportive behaviors within their organizational environment, which greatly fosters their sense of teaching efficacy. As a vital component of the work environment, inclusive leadership plays a significant role in shaping young teachers’ perceptions of their teaching efficacy. Warner et al. (2011) identified four predictor factors influencing self-efficacy: mastery experience, vicarious experience, verbal persuasion, and physiological state. Inclusive leadership can enhance young teachers’ sense of teaching efficacy by positively influencing the four factors previously outlined. First, inclusive leadership fosters an enriching environment that promotes the professional development of young teachers. Providing opportunities such as research training, expert seminars, teaching observations, and study abroad programs, empowers these young teachers to enhance their teaching experiences, cultivate confidence in their abilities, and strengthen their sense of effectiveness in the classroom (Nguyen et al., 2024). Second, vicarious experiences frequently stem from the exemplary teaching performances of influential and seasoned leaders within their proximity. Young teachers often observe the instructional practices of these leaders, emulating their methods and drawing inspiration to refine their teaching skills. This process enhances their teaching effectiveness and boosts their sense of teaching efficacy. Moreover, inclusive leaders promote equitable treatment for young teachers (López-López et al., 2024). When these teachers see their peers being recognized for completing teaching tasks, they believe their efforts can similarly lead to positive outcomes. This belief further strengthens their sense of teaching efficacy. Third, inclusive leadership recognizes the teaching potential of young teachers and actively extends trust and encouragement to them. This support greatly enhances their confidence and self-perception as they embrace their teaching responsibilities. Furthermore, inclusive leadership promotes open discussions about educational objectives with these teachers, empowering them to strive toward achieving these goals. Consequently, young teachers come to view their roles as meaningful, fostering the development of a positive self-concept. Fourth, as a people-oriented and positive leadership style, inclusiveness significantly enhances the psychological well-being of young teachers through constructive communication and emotional connection (Specht et al., 2016). Inclusive leadership engages with young teachers fairly and equitably, fostering supportive relationships that transcend traditional hierarchical distinctions between “superiors and subordinates.” This nurturing environment encourages emotional exchanges, cultivates a sense of security and trust, and empowers young teachers to adopt an optimistic mindset, ultimately bolstering their teaching efficacy (Edinger and Edinger, 2018). Hence, we hypothesize the following:

*H2*: Inclusive leadership positively relates to young teachers’ teaching efficacy.

### 2.3 The mediating role of young teachers’ teaching efficacy

The socially embedded model of thriving suggests that workplace characteristics promote individual motivational behaviors, ultimately enhancing work thriving (Spreitzer et al., 2005). One key situational characteristic is inclusive leadership, which plays a significant role in shaping employees’ attitudes toward their work and, in turn, influences their thriving (Younas et al., 2023). Social cognitive theory suggests self-efficacy may function as a mechanism through which the external environment influences individual behavioral choices (Bandura, 1991). Teaching efficacy, a vital aspect of teachers’ positive psychological capital, embodies their confidence in their teaching skills. Yong teachers with high teaching efficacy are more likely to appreciate and embrace the open, approachable, and supportive qualities that characterize inclusive leadership. Leveraging the principle of reciprocity, these young teachers are motivated to enhance their engagement and enthusiasm in their teaching roles to reciprocate the organizational support they receive. This increased level of commitment fosters better learning behaviors that emphasize skill development. Therefore, this paper proposes that teachers’ teaching efficacy mediate the relationship between inclusive leadership and thriving at work. First, inclusive leadership demonstrates a remarkable openness (Umrani et al., 2024), valuing young teachers’ teaching abilities and contributions. It encourages them to embrace scientific research, engage in experimentation, and actively participate in learning and educational practice. This approach fosters a stronger sense of self-worth in young teachers, enhancing their teaching efficacy. When young teachers feel confident in their capabilities, they are more inclined to approach their responsibilities enthusiastically and positively (Moè et al., 2010). Second, when inclusive leadership creates an environment that embraces young teachers’ teaching deficiencies and mistakes, it greatly enhances their psychological safety and teaching efficacy (Carmeli et al., 2010; Hirak et al., 2012). Therefore, these teachers are more inclined to take the initiative to fulfill their organizational responsibilities. Importantly, an increased sense of teaching efficacy can further foster the autonomy and effectiveness of young teachers. Consequently, when they feel supported and encouraged by their leaders, they are more likely to engage actively in their teaching efforts and commit to continuous learning and improvement. Third, inclusive leadership is essential in enhancing the teaching competence of young teachers by providing substantial support and resources. This enhancement elevates teachers’ confidence and cultivates a stronger sense of teaching efficacy. Young teachers experience a revitalized sense of vitality when their self-efficacy grows. Grounded in the conservation of resources theory, we observe that these teachers are motivated to actively seek out opportunities for professional development to maintain their confidence, and ultimately contribute to thriving. Finally, inclusive leaders are committed to nurturing high-quality relationships with young teachers (Óskarsdóttir et al., 2020). They actively listen to young teachers’ suggestions, show genuine concern for their teaching efforts, and maintain strong accessibility. This dedication enhances the transparency of the relationship be-tween leaders and young teachers, fostering a positive organizational environment that encourages young teachers’ professional growth and self-development, and bolstering their motivation to learn. Hence, we hypothesize the following:

*H3*: Young teachers’ teaching efficacy will mediate the relationship between inclusive leadership and thriving at work.

### 2.4 The moderating role of professional identity

Professional identity denotes the integration of an individual’s cognitive affirmation, affective engagement, and behavioral disposition toward their profession and internalized professional roles (Çoban, 2022), encompassing four dimensions: occupational values, role values, occupational belonging, and occupational behavioral inclination (Xu et al., 2023; Han et al., 2022). For young college teachers, professional identity is a dynamic construct through which they negotiate and establish their professional roles via ongoing engagement in pedagogical practices, research activities, and institutional interactions (Flores, 2020), involving continuous self-reflection and contextual adaptation to reconcile personal aspirations with disciplinary and institutional expectations (Beijaard et al., 2004). Existing research indicates that young teachers’ professional identity positively influences their teaching efficacy by enhancing professional confidence (Xiaoyan, 2023), elevating teacher-student interaction quality (Skinner et al., 2021), stimulating pedagogical innovation awareness (Dickinson et al., 2022), and deepening teaching responsibility. Drawing on social cognitive theory, individuals interpret environmental stimuli through cognitive schemas and modulate behavior via goal-setting (Caprara et al., 2013). In this frame-work, inclusive leadership acts as an external cue, whereas professional identity serves as an internal trait. Their interaction facilitates transforming extrinsic motivations into intrinsic drivers, prompting self-regulated adaptation that enhances well-being and teaching practices.

First, young teachers with a strong professional identity often view inclusive leadership behaviors as essential for their professional development and career aspirations. Research indicates that inclusive leadership fosters a positive school culture, which enhances teachers’ job satisfaction and retention (Gómez-Hurtado et al., 2021). For instance, they perceive leader-facilitated interdisciplinary learning as opportunities for professional growth rather than as burdens. Consequently, they proactively adjust their teaching objectives, integrate cross-disciplinary knowledge, and utilize favorable environmental factors to enhance their teaching efficacy. Additionally, young teachers perceive interdisciplinary learning as an opportunity for growth rather than a burden. They actively seek to integrate knowledge across disciplines, which not only enhances their teaching efficacy but also aligns with their cognitive frameworks regarding professional development. Second, inclusive leadership significantly enhances young teachers’ professional identity and teaching efficacy by fostering a supportive environment that encourages innovative teaching practices. Research indicates that teachers with high professional identity, particularly in inclusive settings, engage more actively in professional development and adopt inclusive pedagogical strategies (Steinert et al., 2019), such as cooperative learning and flipped classrooms, which lead to improved student engagement and academic performance. Furthermore, the implementation of interactive teaching methods has been shown to promote critical thinking and collaboration among students, thereby enhancing their learning outcomes. Third, the relationship between young teachers with a strong professional identity and inclusive leadership can be conceptualized as an implicit “career development contract.” This contract suggests that these teachers perceive the support from inclusive leadership and the enhancement of their teaching abilities as mutually beneficial. Specifically, young teachers who possess a high professional identity view the improvement of their teaching effectiveness as a personal responsibility, believing that “I have the responsibility to improve teaching effectiveness.” This ongoing self-regulation fosters a sustained impact of inclusive leadership support, reinforcing the psychological benefits derived from their professional identity. Hence, we hypothesize the following:

*H4*: Professional identity positively moderates the relationship between inclusive leader-ship and teaching efficacy.

Young teachers who possess a strong professional identity exhibit a profound alignment with the fundamental principles of the teaching profession, including the commitment to education and the pursuit of professional development. They are more likely to internalize the support provided by inclusive leadership, such as the provision of teaching autonomy, facilitation of personalized development, and opportunities for democratic participation (Huy and Phuc, 2024), as motivational factors that enhance their teaching capabilities. This internalization significantly amplifies the positive impact of inclusive leadership on teaching efficacy. For example, young teachers with a robust professional identity actively translate instructional resources offered by leaders into effective classroom practices, thereby reinforcing their self-efficacy beliefs regarding effective teaching competence. In contrast, those with a weaker professional identity may view such leadership support as externally imposed obligations rather than intrinsic motivators. Furthermore, young teachers with strong professional identity tend to anchor their teaching self-efficacy in professional ideals (Chen et al., 2023), framing efficacy enhancement as a pathway to realize both self and social value. They translate this efficacy belief into sustained work engagement, for example, by proactively optimizing teaching designs and deepening teacher-student interaction, thereby fostering vigorous, dedicated, and focused thriving at work (Bjerke and Xenofontos, 2024). This suggests that a strong professional identity not only strengthens the relationship between inclusive leadership and teaching efficacy but also significantly impact their teaching efficacy into thriving at work. Collectively, these indicate that professional identity significantly moderates the mediating role of teaching efficacy in the relationship between inclusive leadership and thriving at work. Hence, we hypothesize the following.

*H5*: Professional identity positively moderates the mediating role of teaching efficacy be-tween inclusive leadership and thriving at work.

**Figure 1:**
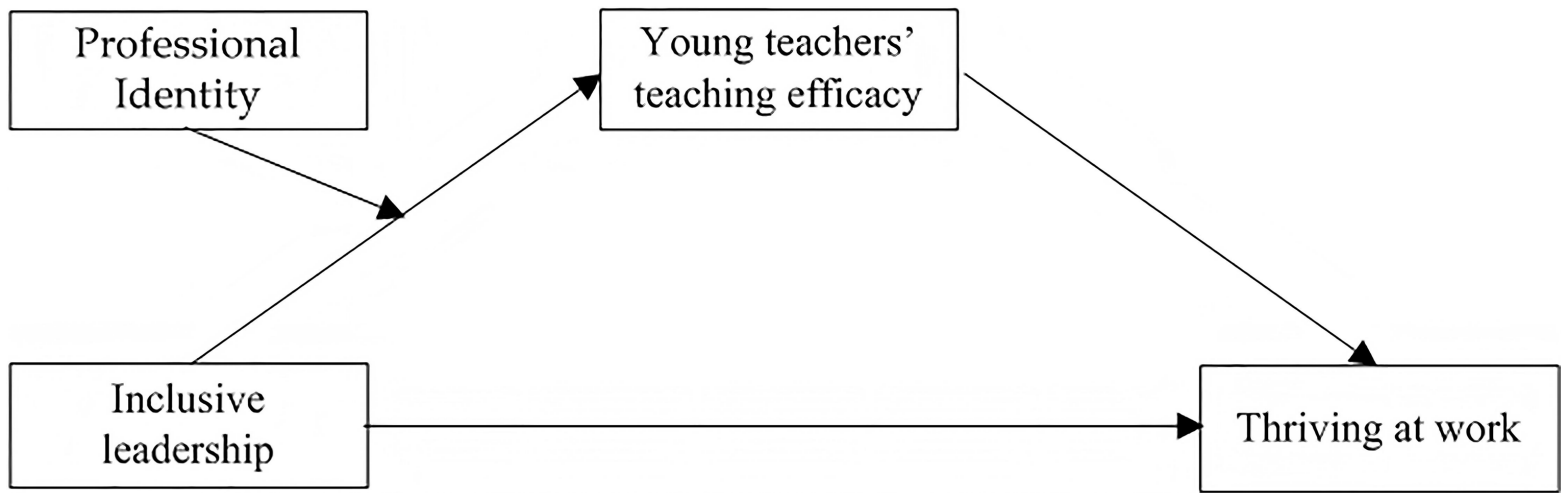
Basic conceptual framework.

As mentioned above, we proposed a conceptual research framework (see Figure 1).

## 3 Materials and methods

### 3.1 Participants and procedures

We collected information from the official websites of various universities throughout China to identify young faculty members under 40. We adopted a 40-year age limit to define our sample, justified by its dual significance in policy and theory. This stage is not only critical for the professional maturation of young academics but also mirrors the formal standards of youth talent programs in China (McGovern and Barry, 2000; Lian et al., 2021). Moreover, maintaining this threshold facilitates cross-study comparability, as it resonates with the conventional age-based categorization in the field (Sloan and LaPlante, 2002; Goodson et al., 2006; Bhatnagar and Das, 2014). Subsequently, we emailed them a link to an online questionnaire and a QR code generated by an online platform named “WenJuanXing” for data collection. The respondents comprised a diverse cohort from seven provinces, including Beijing, Shanghai, Shanxi, Hubei, Henan, Shandong and Xinjiang. They were associated with 20 universities in first-tier, second-tier, and third-tier cities. Specifically, the sample included five universities in Beijing, four in Shanghai, three each in Shanxi and Hubei, two each in Henan and Shandong, and one in Xinjiang. All participants were fully informed about the study and gave their consent. Data collection was conducted anonymously to safeguard respondent privacy and minimize the potential for social desirability bias. A total of 800 questionnaires were distributed. After excluding invalid submissions due to missing data or uniform responses, we retained 755 valid questionnaires, yielding an effective response rate of 94.38%. As shown in Table 1, among the 755 valid responses, females comprised 54.171% of the sample, and approximately 65.166% of respondents were aged between 30 and 40 years. Those with work experience ranging from 4 to 6 years comprised 43.046% of the sample. Among the participants, 43.046% of lecturer/assistant professor, 36.556% of associate professor, and 56.821% of the faculty hold a doctorate degree.

**Table 1:** Demographic information.

Categories	*N*
Complete sample	755 (100%)
Gender (Gend)	
Male	346 (45.828%)
Female	409 (54.171%)
Age (Age)	
Less than 30 years	263 (34.834%)
30–40 years	492 (65.166%)
Teaching experience (Expe)	
Less than 3 years	239 (31.656%)
4–6 years	325 (43.046%)
Higher than 7 years	191 (25.298%)
Professional title (Title)	
Junior/Assistant Lecturer	103 (13.642%)
Lecturer/Assistant Professor	325 (43.046%)
Associate Professor	276 (36.556%)
Professor	51 (6.755%)
Educational attainment (Edu)	
Bachelor’s degree	69 (9.139%)
Master‘s degree	201 (26.623%)
PhD	429 (56.821%)
PhD candidate	56 (7.417%)

*N* = 755. IL, inclusive leadership; TE, teaching efficacy; PI, professional identity; TAW, thriving at work.

### 3.2 Measurements

#### 3.2.1 Inclusive leadership

This study used the measurement scale developed by Carmeli et al. (2010). This scale comprises three dimensions: openness, effectiveness, and accessibility, totaling nine items. Notable examples of these items include statements such as, “My leader is receptive to new ideas,” “My leader actively engages in discussions about challenges with me,” and “My leader encourages me to approach him/her when I encounter new problems.” The questions were scored using a 5-point Likert scale, ranging from 1 to 5, with 1 representing “strongly disagree” and 5 signifying “strongly agree.” The scale shows good reliability, the Cronbach *α* coefficient of the questionnaire was 0.829, and the KMO was 0.908.

#### 3.2.2 Thriving at work

The thriving at work scale employed an instrument developed by Porath et al. (2012), which includes two dimensions of the construct: learning and vitality. The learning dimension contains 5 items, with representative statements such as “I continue to learn more as time goes by.” The vitality dimension also consists of 5 items, featuring representative statements like “I feel alive and vital”. The response for thriving at work is on a 5-point Likert scale (1 = strongly disagree, 5 = strongly agree). The Cronbach’s α for this construct was 0.807, and the KMO was 0.899.

#### 3.2.3 Teaching efficacy

The teachers’ teaching efficacy Questionnaire, was developed by Wu and Chim (2017). This questionnaire included 12 items that examined the teacher’s idea about his effective control over instructional strategies and classroom management. It used a 5-point Likert scale, ranging from 1 (strongly disagree) to 5 (strongly agree), to rank the teachers’ teaching efficacy. Higher scores reflect a greater sense of teaching efficacy. The reliability estimates of the questionnaire were calculated using Cronbach’s alpha. The questionnaire’s reliability coefficient was 0.833, the KMO was 0.918, showing a reasonably acceptable index of reliability coefficient.

#### 3.2.4 Professional identity

Teachers’ professional identity was measured using the Teachers’ Professional Identity Scale (Li et al., 2025; Li et al., 2023), which comprises four dimensions: occupational values, role values, the sense of occupational belonging and professional behavior inclination, with a total of 18 items (e.g., I am proud of being a teacher). Using the five-point Likert scale, with a higher score conveying a strong sense of professional identity. The scale was proved to have good validity, with Cronbach’s alpha coefficient of 0.928 observed in our study, and the KMO was 0.972.

#### 3.2.5 Control variables

Five control variables (the teachers’ gender, age, teaching experience, professional title, and education) were used in this analysis to control the possibility that certain demographic factors may influence thriving at work. Gender (Gend) is a dummy variable coded 1 for males and 0 for females. Age (Age) was categorized into two groups: 1 for individuals aged 30 and below, 0 for aged 30–39. Years of teaching experience (Expe) are classified as 1 for less than 3 years, 2 for 4–6 years, and 3 for 7 years and above. Professional titles (Title) were categorized into four groups: 1 for junior/assistant lecturer, 2 for lecturer/assistant professor, 3 for associate professor, 4 for professor. Educational attainment (Edu) was classified into four levels: 1 for bachelor’s degree, 2 for master’s degree, 3 for PhD, 4 for PhD candidate.

### 3.3 Data analysis

Statistical analyses were performed using SPSS 26.0 and AMOS 29.0. First, AMOS 29.0 was employed to conduct confirmatory factor analysis (CFA), and SPSS 26.0 was used for data entry and analysis. Second, we performed descriptive statistics, and correlation analyses. Third, the PROCESS macro for SPSS was used to test the mediating role of teaching efficacy in the relationship between inclusive leadership and thriving at work, with Bootstrap (5,000 resamples) employed to validate the indirect effects. Finally, moderated regression analysis incorporating interaction terms was utilized to examine the moderating role of professional identity.

## 4 Results

### 4.1 Common method Bias test

This study utilizes self-reported data collection, which may have introduced common method bias. To mitigate this potential issue, we ensure that data collection is conducted anonymously and employ both forward and reverse scoring techniques to enhance the objectivity of our findings and minimize bias. To assess the presence of common method bias in our sample, we perform Harman’s single-factor test. The results indicate that four factors had eigenvalues above 1, with the highest variance ac-counted for by a single factor at 31.963%, falling below the commonly accepted thresh-old of 40%. This suggests that significant common method bias is not a concern in our data.

### 4.2 Reliability and validity testing

Prior to hypothesis testing, the adequacy of the measurement model was rigorously evaluated. A four-factor baseline model was first specified according to the theoretical framework, followed by the construction of nested three-factor, two-factor, and single-factor models for comparative assessment (see Table 2). Comparative analysis of model fit indices revealed that the four-factor baseline model demonstrated significantly better fit statistics (*χ*^2^/df = 2.843, RMSEA = 0.042, IFI = 0.965, CFI = 0.965, TLI = 0.961) compared to all models. These findings afford robust evidence of strong discriminant validity among the primary variables, demonstrating that the research data withstood the rigorous scrutiny of confirmatory factor analysis (CFA) and sup-porting the construct validity of the measurement instruments.

**Table 2:** Results of confirmatory factor analyses.

Model	*χ*^2^/df	RMSEA	IFI	CFI	TLI
Four-factor model:	2.843	0.042	0.965	0.965	0.961
IL, TE, PI, TAW					
Three-factor model:	5.296	0.061	0.902	0.901	0.893
IL, TE + PI, TAW					
Two-factor model:	9.831	0.091	0.782	0.781	0768
IL + TAW, TE + PI					
Single-factor model:	15.742	0.122	0.634	0.633	0.615
IL + TE + PI + TAW					

### 4.3 Descriptive statistics and correlation analysis

Table 3 presents the descriptive statistics and correlations for each variable. The mean value of Gend is 0.458, and the standard error is 0.499, indicating that female teachers outnumber their male counterparts. The average Age is 0.348, with a standard deviation of 0.477, suggesting that most young teachers fall within the 30–40 age range. The average years of Expe are 1.936, with a standard deviation of 0.752. This indicates that most young teachers possess between 4 and 6 years of experience in the field. The means of Title and Edu were 2.364 and 2.625, respectively, indicating that the majority of young teachers held the professional title of lecturer/assistant professor and possessed doctoral degrees. Correlational analyses indicated that inclusive leadership correlated significantly with both thriving at work (*r* = 0.500, *p* < 0.01) and teaching efficacy (*r* = 0.267, *p* < 0.01), while teaching efficacy was positively associated with thriving at work (*r* = 0.475, *p* < 0.01). These findings supported Hypotheses 1 and 2, laying the groundwork for subsequent mediational tests.

**Table 3:** Descriptive statistics and Pearson correlation matrix.

Variables	Mean	Sd	1	2	3	4	5	6	7	8	9
Gend	0.458	0.499	1								
Age	0.348	0.477	−0.025	1							
Expe	1.936	0.752	−0.053	−0.023	1						
Title	2.364	0.8	−0.017	0.084*	0.005	1					
Edu	2.625	0.752	0.073*	−0.072*	−0.007	−0.004	1				
Thriving at Work	4.386	0.439	0.059	−0.049	−0.136**	−0.076*	0.037	1			
Inclusive Leadership	4.527	0.499	0.062*	−0.059	−0.158**	−0.043	0.054	0.500**	1		
Teaching Efficacy	4.43	0.693	0.016	0.011	−0.052	−0.063	0	0.475**	0.267**	1	
Professional Identity	4.309	0.89	0.068*	−0.027	−0.178**	−0.025	0.037	0.401**	0.238**	0.211**	1

### 4.4 Hypothesis testing

#### 4.4.1 Testing the mediation model

This study adopts the Baron and Kenny (1986) approach to test the mediating effect of teaching efficacy. First, we examined the influence of the independent variables on the dependent variables. As shown in Model 3 of Table 4, inclusive leadership significantly positively impacts thriving at work (*β* = 0.427, *p* < 0.001), supporting Hypothesis 1. Second, we examined the influence of independent variables on the mediating variables. As shown in Model 1 of Table 4, inclusive leadership exhibits a significant positive impact on teaching efficacy (*β* = 0.369, *p* < 0.001), supporting Hypothesis 2. And third, we examined the effects of both independent and mediating variables on the dependent variable. We incorporated teaching efficacy into the regression analysis in Model 5. The results indicate that teaching efficacy has a significant positive impact on thriving at work (*β* = 0.232, *p* < 0.001). Additionally, the influence of inclusive leader-ship on thriving at work decreases (from 0.427*** to 0.341***), suggesting that teaching efficacy partially mediates the relationship between inclusive leadership and thriving at work, supporting Hypothesis 3.

**Table 4:** Regression results.

Variables	Teaching efficacy		Thriving at work		
	Model 1	Model 2	Model 3	Model 4	Model 5
Inclusive leadership	0.369***	0.330***	0.427***		0.341***
	(7.41)	(6.66)	(15.13)		(12.79)
Teaching efficacy				0.295***	0.232***
				(14.64)	(12.25)
Professional Identity		0.125***			
		(4.50)			
Inclusive leadership × Professional identity		0.277***			
		(5.04)			
Gend/Age/Expe/Tiltle/Edu	Yes	Yes	Yes	Yes	Yes
_cons	2.905***	4.520***	2.572***	3.209***	1.898***
	(10.79)	(34.61)	(16.84)	(26.56)	(12.66)
*N*	755	755	755	755	755
*F*	10.097	13.674	43.142	40.631	65.772
*R* ^2^	0.075	0.128	0.257	0.246	0.381
*R*^2^_a	0.068	0.119	0.251	0.240	0.376

*N* = 755. *p < 0.05, **p < 0.01, ***p < 0.001.

This study also utilized the SPSS 26.0 PROCESS macro to examine the mediating effect of teaching efficacy. We generated 5,000 bootstrap samples and 95% confidence intervals (CIs). As presented in Table 5, the Bootstrap-estimated direct effect was 0.341 (95% CI: 0.290–0.392), confirming its significance. The mediation effect was 0.086 (95% CI: 0.062–0.112), both intervals excluding zero, thus supporting the significance of the mediation effect and validating Hypothesis 3.

**Table 5:** Bootstrap mediation effect tests.

Condition	Effect	SE	LLCI	ULCI
Total Effect	0.427	0.028	0.373	0.481
Direct Effect	0.341	0.026	0.290	0.392
Indirect Effect	0.086	0.013	0.062	0.112

*N* = 755. Number of bootstrapping iterations = 5000.

#### 4.4.2 Testing the moderated mediation model

**Figure 2:**
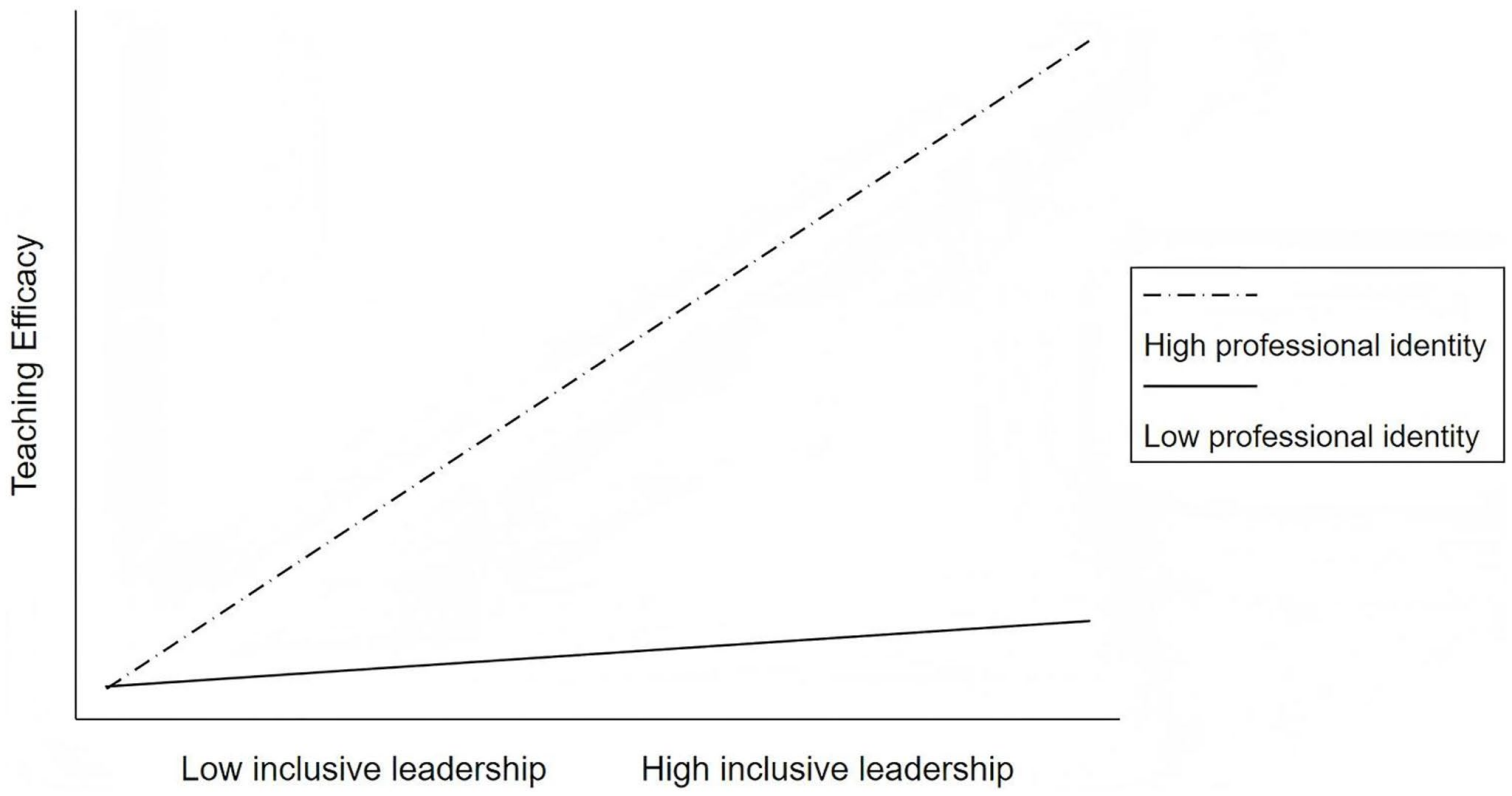
Moderation effect of inclusive leadership and professional identity on teaching efficacy.

To mitigate multicollinearity issues before testing the moderating effect, inclusive leadership and professional identity were mean-centered to derive their interaction term. This interaction term was incorporated into the regression model, with results presented in Table 4. Model 2 showed that inclusive leadership significantly predicted teaching efficacy (*β* = 0.330, *p* < 0.001). Critically, the interaction term between inclusive leadership and professional identity was significantly positively associated with teaching efficacy (*β* = 0.277, *p* < 0.001), lending empirical support to Hypothesis 4. As illustrated in Figure 2, the positive association between inclusive leadership and teaching efficacy was significantly enhanced at higher levels of professional identity.

To investigate whether young teachers’ professional identity moderates the indirect effect of teaching efficacy in the relationship between inclusive leadership and thriving at work, we employed the Process 4.1 macro for SPSS 26.0 to compute un-standardized conditional indirect effects at high (mean +1 SD) and low (mean −1 SD) moderator values. As presented in Table 6, when professional identity was high, inclusive leadership had a significant indirect effect on thriving at work through teaching efficacy (effect = 0.490, bootstrap SE = 0.032, 95% CI [0.427, 0.553]). At low professional identity, the indirect effect was weaker but remained significant (effect = 0.103, boot-strap SE = 0.033, 95% CI [0.038, 0.169]). The significant difference between these conditional indirect effects supports the existence of a moderated mediation effect, thereby validating Hypothesis H5.

**Table 6:** Moderated mediation effect test results.

Variable	Effect	SE	LLCI	ULCI
Mean −1 SD	0.103	0.033	0.038	0.169
Mean +1 SD	0.490	0.032	0.427	0.553

*N* = 755. Number of bootstrapping iterations = 5000.

## 5 Discussion

### 5.1 Theoretical contribution

First, this study serves as an empirical validation of an established Western construct within the Chinese higher education context. Prior research has largely relied on Western definitions and scales (Carmeli et al., 2010; Hollander, 2012), largely overlooking China’s unique cultural context. The study investigates the effects of inclusive leadership, testing its cross-cultural applicability, enriching indigenous theoretical development, and revealing its distinctive mechanisms within universities. The findings offer theoretical insights and practical pathways for developing culturally attuned management practices. Furthermore, this study enriches the conceptualization of inclusive leadership by elucidating its unique “fault-tolerant” characteristic within the Chinese context. This study enriches the connotation of inclusive leadership within the Chinese context by identifying its “fault tolerance” characteristic, thereby enriching its connotation and complementing the Western framework centered on “openness,” “accessibility,” and “participation.” A leader’s tolerance for failure not only serves as emotional support but also conveys a non-punitive attitude toward setbacks (Zhang and Zhao, 2024). This dual effect helps to alleviate young teachers’ concerns about trial and error and professional risks, strengthens their trust in leaders, and ultimately enhances their teaching and research motivation. However, given that inclusiveness is a social construct, its connotation is inevitably subject to cultural variation, which may profoundly affect young teachers’ comprehension and interpretation of the scale items. Young Chinese teachers conceptualize inclusive leadership primarily through the lens of ‘fault tolerance.’ Distinct from the Western emphasis on discursive equality, this orientation prioritizes a supportive environment where leaders mitigate the repercussions of procedural lapses and provide reparative guidance, thereby bolstering teachers’ psychological safety (Bao, 2024; Lu et al., 2024). Therefore, incorporating “fault tolerance” into the theoretical construction of inclusive leadership serves to eliminate cultural presupposition bias, enhance the theory’s explanatory power and applicability within the Chinese context, and underscore both the necessity and practical value of developing localized scales. Furthermore, it provides Chinese scholars with a robust theoretical foundation for such scale development.

Second, this study elucidates the pivotal impact of inclusive leadership on the teaching efficacy and thriving at work among young faculty, thereby empirically demonstrating its cross-cultural salience and effectiveness within the Chinese organizational context. On the one hand, extant research on the outcomes of inclusive leadership has been predominantly situated within Western cultural frameworks. Given the profound contextual disparities, the cross-cultural generalizability and suitability of inclusive leadership within the Chinese organizational milieu necessitate rigorous empirical validation. Through a targeted survey of young faculty in Chinese universities, this paper substantiates that inclusive leadership significantly bolsters both teaching efficacy and thriving at work, thereby validating the construct’s contextual applicability to organizational management in China. As a nascent leadership construct, inclusive leadership has primarily been explored through theoretical lenses regarding its impact on young faculty. However, direct empirical scrutiny remains notably scarce. By subjecting inclusive leadership to rigorous empirical scrutiny within the Chinese organizational milieu, this study effectively bridges the existing research gap. It not only advances the scholarship on inclusive leadership in non-Western settings but also fosters a critical synthesis between Western theoretical frameworks and Chinese management praxis. On the other hand, this study uncovers the underlying mechanisms through which inclusive leadership influences young teachers’ thriving at work. Previous studies have primarily examined the drivers of thriving at work from the perspectives of psychological capital (Paterson et al., 2014), pressures and demands (Prem et al., 2017), and work support (Zhai et al., 2020), often neglecting the crucial role of teaching efficacy. Anchoring its analysis in Social Cognitive Theory, this paper innovatively identifies teaching efficacy as a central mechanism that shapes positive occupational outcomes for young faculty. Our findings reveal that teaching efficacy serves as a critical intervening pathway between inclusive leadership and workplace thriving, a result that aligns closely with the assertions of Wei et al. (2025), who underscore the role of self-efficacy as a primary driver of professional vitality.

Third, this study theoretically contextualizes teacher professional identity within the Chinese cultural paradigm, offering a nuanced perspective on the boundary conditions of leadership effectiveness. Our findings demonstrate a moderated mediation effect: a robust professional identity amplifies the indirect influence of inclusive leadership on professional prosperity, a process contingent upon the cultivation of teaching self-efficacy. In the Chinese ethos, where educators are mandated to ‘impart knowledge and resolve doubts,’ this culturally-embedded identity serves as a cognitive filter. It dictates how early-career teachers decode leadership signals, navigate their professional roles, and pursue self-actualization. By integrating professional identity as a pivotal moderator, this research elucidates the localized logic through which inclusive leadership functions in Chinese organizational settings.

### 5.2 Practical implications

This study unravels the “black box” of inclusive leadership, illuminating its significant influence on young teachers’ thriving at work. It offers actionable insights for Chinese higher education institutions and policymakers aimed at cultivating a more supportive and inclusive work environment within the distinctive context of China’s higher education system.

First, universities should focus on selecting and cultivating inclusive leaders. Research consistently demonstrates that inclusive leadership significantly enhances young teachers’ thriving at work. Therefore, Chinese universities need to concentrate on nurturing and strengthening inclusive leadership competencies. When recruiting academic leaders and department heads, higher education institutions should prioritize inclusive leadership as a key criterion in their selection process. It’s essential to evaluate whether candidates demonstrate respect and equity when interacting with young teachers, and can provide a supportive environment where junior educators can grow and learn from their mistakes without fear of retribution or bias. By embracing inclusive leadership, universities can cultivate a positive, growth-oriented culture that benefits both their educators and students. Within the Chinese higher education context, where hierarchical structures and centralized governance remain prevalent, it is particularly important to establish an inclusive organizational structure that fosters a collaborative environment in which diverse ideas can flourish. To achieve this vision, it is essential to cultivate an institutional culture that prioritizes inclusivity. Additionally, it is important to encourage academic leaders to adopt inclusive practices by integrating these behaviors into their performance evaluation criteria, a mechanism that aligns with China’s ongoing university governance reforms. This approach will motivate leaders to actively advocate for inclusivity as a fundamental value in their work. By soliciting and incorporating feedback from these educators, leaders can refine and strengthen their inclusive leadership capabilities, ultimately fostering a more supportive and inclusive work environment in Chinese universities.

Second, universities should prioritize enhancing and cultivating young teachers’ teaching efficacy. Teaching efficacy refers to teachers’ beliefs and self-assessments regarding their ability to effectively carry out specific teaching tasks and demonstrate the necessary pedagogical skills. Our research shows that inclusive leadership significantly enhances young teachers’ teaching efficacy, and contributes to their thriving at work. Therefore, Chinese universities should improve young teachers’ teaching efficacy through inclusive leadership. Leaders must prioritize the development of young teachers’ competencies and overall skills, while also improving the quality of teaching training by implementing a robust, well-structured training system that aligns with China’s national initiatives for teacher development, such as the “Excellent Teacher Training Program”. This approach will ensure their long-term growth, success, and commitment to the profession. Universities should aim for effective “person-job matching” by offering young teachers more opportunities for teaching and research within the framework of China’s “Double First-Class” construction, which emphasizes the integration of teaching excellence and research innovation. This strategy will improve the quality of young educators and cultivate a stronger sense of efficacy in their teaching roles. Moreover, leaders should foster equitable and supportive relationships with young teachers, promoting open dialog, active listening, and effective communication to create a collaborative and inclusive environment in Chinese academic departments. Leaders should cultivate an environment of open communication with young teachers, dedicating time to genuinely understanding the challenges they face in the context of China’s highly competitive academic labor market and the pressures associated with promotion reviews. By providing constructive feedback and assisting them in analyzing the underlying causes of their difficulties, leaders can effectively guide these educators in overcoming setbacks. This supportive approach can boost their self-confidence and enhance their sense of teaching efficacy.

Finally, this study reveals that professional identity moderates the relationships between inclusive leadership and teaching efficacy, as well as inclusive leadership and thriving at work. This underscores the need to prioritize professional identity cultivation among young teachers in China. At the national level, governments should prioritize policies to enhance young teachers’ social prestige and economic security, such as increasing educational investment and optimizing salaries, to underscore education’s societal value and professional status. At the societal level, communities should foster a culture of teacher respect and educational prioritization via official media platforms and public advocacy campaigns, strengthening young teachers’ professional pride. At the institutional level, Chinese universities should enhance participatory governance mechanisms to cultivate institutional ownership among young teachers, boosting their sense of belonging and accountability within the evolving landscape of university governance reforms in China.

### 5.3 Limitations and future research

Despite the contributions made by this paper, several drawbacks and areas need to be addressed in future research.

First, this study employs an online questionnaire survey to mitigate the common method bias typically associated with cross-sectional data, collecting responses from current college teachers and alumni. Nevertheless, the loss of college data samples leads to a partially non-random sample, which may compromise the external validity of the findings. Future research could improve questionnaire design by incorporating flexible timing and personalized reminders, potentially leading to a more accurate assessment of young teachers’ teaching efficacy and thriving at work. Additionally, expanding the research sample to include a wider range of industries and employee demographics could significantly enhance the validity of the findings.

Second, although this study reveals that inclusive leadership affects young teachers’ thriving at work, it lacks sufficient data to substantiate the impact of thriving at work on teaching performance. Future research can further explore the relationship between thriving at work and teaching performance by investigating outcome variables that effectively capture changes in teaching performance. This approach will deepen our understanding of how the young teachers’ thriving at work impacts their teaching performance, ultimately leading to more tailored recommendations for the sustainable development of education. Meanwhile, this study examined the role of inclusive leadership in promoting young teachers’ thriving at work, informed by social cognitive theory and the social embeddedness model of thriving at work. However, this paper focuses solely on the mediating role of teaching efficacy, which limits its ability to fully elucidate the underlying mechanisms through which inclusive leadership promotes thriving at work. Future research could delve deeper into the various mediating mechanisms through which inclusive leadership impacts young teachers’ thriving at work. Potential factors to examine include perceived organizational support, organizational error-management climate, basic psychological needs, and supervisor develop-mental feedback. Further-more, specific traits of teachers, such as a strong internal locus of control, may act as moderating variables that increase the probability of achieving thriving at work. Investigating these elements will enhance the theoretical frame-work of current research and also enrich our understanding of the interplay between inclusive leadership and young teachers’ thriving at work.

Third, although this study employs an established Western scale to assess inclusive leadership, the extent to which its dimensions and items capture the unique cultural nuances of inclusion within Chinese organizations warrants further investigation. Given that cultural backgrounds profoundly shape leaders’ managerial philosophies, cognitive schemas, and behavioral manifestations, it is imperative to develop culturally adaptive instruments to advance indigenous research. Accordingly, future research should prioritize the development of an indigenous inclusive leadership scale grounded in the Chinese cultural context, with a specific focus on the unique professional experiences of young university faculty. Developing a Chinese-specific inclusive leadership scale within the higher education sector would not only enhance young faculty members’ conceptualization of inclusion, but also elucidate its underlying structural dimensions and operative mechanisms, thereby significantly enriching the extant literature on inclusive leadership.

Fourth, a common methodological concern in leadership research is referent ambiguity, specifically whom respondents consider “the leader.” In Chinese universities, faculty navigate multiple authority layers, from department chairs to deans, whose leadership styles may differ. Although we instructed participants to focus on their immediate supervisors, some may have anchored responses on more distal leaders. This ambiguity introduces potential measurement bias, representing a limitation of this study. Future research should explicitly specify the leadership referent (e.g., “your department head”) and employ multi-wave or multi-source designs to enhance referent clarity and causal inference.

## 6 Conclusion

This study, rooted in the social embeddedness model of thriving at work and social cognitive theory, empirically tests the influence of inclusive leadership on the thriving at work of young teachers, with a particular focus on examining the mediating role of teaching efficacy and the moderating role of professional identity. Based on an analysis of 755 valid questionnaires, the study yields the following key findings: First, inclusive leadership has a profoundly positive impact on young teachers’ thriving at work. By fostering a supportive and inclusive environment, leaders empower these educators to take an active role in decision-making processes, cultivating a sense of autonomy and openness that not only sparks their interest but also deepens their commitment to their teaching roles. Moreover, inclusive leadership acknowledges and values young teachers’ significant contributions to education, strengthening their sense of belonging and professional identity. Ultimately, this convergence of factors substantially enhances the overall workplace satisfaction and prosperity of young educators, creating a positive and thriving work environment. Second, inclusive leadership profoundly enhances young teachers’ teaching efficacy. By offering programs such as teaching training, seminars, and study abroad opportunities, inclusive leadership fosters the professional development and overall experience of young teachers. Moreover, it creates a supportive environment characterized by fairness, equality, trust, and encouragement. This nurturing atmosphere is essential in significantly boosting young teachers’ confidence as they embrace their teaching responsibilities. Third, teaching efficacy serves as a mediator in the relationship between inclusive leadership and thriving at work. Inclusive leadership cultivates a supportive environment that acknowledges and values the teaching abilities of emerging teachers. It fosters robust interpersonal relationships with these teachers and encourages their engagement in scientific research. As a result, young teachers experience an increased sense of teach-ing efficacy, which subsequently boosts their motivation and enthusiasm for learning. Fourth, professional identity significantly moderates the positive association between inclusive leadership and teaching efficacy, such that stronger professional identity amplifies the positive impact of inclusive leadership on teaching efficacy. Additionally, professional identity further moderates the indirect effect of inclusive leadership on thriving at work via teaching efficacy, higher professional identity strengthens the in-direct pathway through which inclusive leadership enhances thriving at work me-diated by teaching efficacy.

## Data Availability

The raw data supporting the conclusions of this article will be made available by the authors, without undue reservation.
